# Genetic tumor syndromes in female cancer: insights into inherited cancer predisposition and clinical implications

**DOI:** 10.1007/s00404-025-08270-6

**Published:** 2026-01-13

**Authors:** Annika Krückel, Julia Gocke, Manuel Hörner, Katharina Keller, Carolin Müller, Lena Brückner, Felix Heindl, Carolin C. Hack, Matthias W. Beckmann, Niklas Amann

**Affiliations:** 1 Department of Gynecology and Obstetrics, Universitätsklinikum Erlangen, Comprehensive Cancer Center Erlangen-EMN (CCC ER-EMN), Universitätsstraße 21/23, 91054 Erlangen, Germany; 2 Friedrich-Alexander-Universität Erlangen-Nürnberg (FAU), Erlangen, Germany; 3 Bavarian Cancer Research Center (BZKF), Erlangen, Germany

**Keywords:** Tumor syndromes, Hereditary breast and ovarian cancer, Gynecologic malignancies, Endometrial cancer, Ovarian cancer, Cervical cancer, Breast cancer

## Abstract

A relevant proportion of malignancies predominantly or exclusively affecting women, including breast and gynecologic cancers, is attributable to hereditary tumor syndromes, profoundly impacting cancer risk, prognosis, and therapeutic management. Today, the routine use of comprehensive germline panels has shifted the focus from solely pathogenic *BRCA1/2* variants to include numerous pathogenic variants of other high- and moderate-risk genes. A broad spectrum of genetic alterations has been identified as causative for Hereditary Breast and Ovarian Cancer syndrome (HBOC), encompassing not only *BRCA1* and *BRCA2*, but also *PALB2*, *ATM*, *BARD1*, *CHEK2*, *BRIP1*, *RAD51C*, and *RAD51D*. Beyond HBOC, numerous additional hereditary tumor syndromes are of significance in senologic and/or gynecologic oncology, including Li-Fraumeni syndrome, Lynch syndrome, *DICER1* syndrome, Hereditary Diffuse Gastric Cancer, Neurofibromatosis type 1, Peutz-Jeghers syndrome, *PTEN* hamartoma tumor syndrome, Tuberous Sclerosis, and pathogenic variants in *NBN* and *SMARCA4*. Affected individuals are offered specialized surveillance to enable early detection or even prevention of cancer. In addition to regular clinical examinations and imaging, preventive strategies may include risk-reducing surgery. Pathogenic germline variants also influence therapeutic management of cancer patients. For specific indications, targeted therapies are available, for example PARP [poly (ADP-ribose) polymerase] inhibitors for pathogenic *BRCA* variant carriers across multiple tumor entities. Optimal management requires interdisciplinary coordination, encompassing genetic counseling, early detection, and risk-reducing strategies within specialized centers. This review provides a comprehensive overview of hereditary tumor syndromes predisposing to breast and gynecologic malignancies, with a focus on genetic basis, associated cancer risks, and implications for clinical management. By delineating these syndromes, it aims to assist clinicians in recognizing hereditary cancer predisposition and in guiding affected individuals within routine senologic and gynecologic practice.

## Introduction

A substantial proportion of malignancies predominantly or exclusively affecting women, including breast, ovarian, and endometrial cancers, arise from hereditary tumor syndromes. Advances in germline genetic testing have identified a broad spectrum of pathogenic variants that confer high or moderate lifetime cancer risks in female carriers and are increasingly recognized as critical determinants of prognosis and therapeutic response. Besides Hereditary Breast and Ovarian Cancer (HBOC) and Lynch syndrome, rarer entities such as Peutz-Jeghers syndrome as well as Cowden syndrome are also of particular relevance in oncologic care and cancer prevention among women. In Germany, both breast and gynecologic malignancies are treated within the specialty of gynecology. Consequently, gynecologists play a pivotal role in managing hereditary cancer predisposition. The German Consortium for Familial Breast and Ovarian Cancer (GC-HBOC) represents a nationwide network of certified centers that collaborate to ensure standardized genetic counseling, diagnostics, and evidence-based clinical care. Genetic testing within the consortium is based on the TruRisk^®^ gene panel, which includes *ATM*, *BRCA1*, *BRCA2*, *BARD1*, *BRIP1*, *CDH1*, *CHEK2*, *PALB2*, *PTEN*, *RAD51C*, *RAD51D*, and *TP53* [1]. In addition, the genes *STK11*, *EPCAM*, *MLH1*, *MSH2*, *MSH6*, and *PMS2* are routinely analyzed.

This review presents a structured overview of major hereditary tumor syndromes relevant to cancers affecting predominantly women, outlining their genetic basis, cancer risk profiles, and implications for prevention and individualized care. The discussion primarily follows the genes included in the TruRisk^®^ gene panel of the GC-HBOC and their associated syndromes, while also addressing additional hereditary entities of clinical relevance. By delineating the syndromes that predispose to breast and gynecologic malignancies, it aims to support clinicians in recognizing hereditary cancer risk and guiding affected individuals in everyday gynecologic and senologic practice.

Scope, timing, and target populations of management strategies aimed at mitigating hereditary cancer risk vary across national guidelines and depend on the implicated gene. Additionally, the availability and accessibility of genetic counseling and testing differ considerably across countries and continents [2, 3]. The following sections outline intensified surveillance and preventive strategies from a primarily European perspective, acknowledging that recommendations may differ globally. As this review centers on genes routinely analyzed within the GC-HBOC, a focus on European practice naturally follows, while recognizing that this perspective does not capture the full global diversity of hereditary cancer care.

## Hereditary Breast and Ovarian Cancer syndrome (HBOC)

Hereditary Breast and Ovarian Cancer syndrome (HBOC) is an inherited condition characterized by a significantly elevated risk of breast and ovarian cancers, as well as a possibly increased susceptibility to other malignancies, including pancreatic and — for male carriers — prostate cancers [4]. Approximately 5–10% of breast cancer cases and at least 10–20% of ovarian cancer cases arise from genetic alterations in high- or moderate-risk genes associated with breast and/or ovarian cancer [5–8].

### Pathogenic *BRCA* variants

HBOC is primarily associated with pathogenic variants in *BRCA1* (*FANCS*) and *BRCA2* (*FANCD1*), which are high-penetrance risk genes playing crucial roles in DNA repair mechanisms [1, 4]. Biallelic loss of function leads to severe chromosome instability disorders (Fanconi anemia), characterized by progressive bone marrow failure and heightened predisposition to cancer [9]. In the context of HBOC, the term usually refers to heterozygous carriers of pathogenic *BRCA1* or *BRCA2* variants. The prevalence of pathogenic *BRCA* variants (heterozygote frequency) in the general population is estimated to be around 0.2–0.3%, while certain populations, such as individuals of Ashkenazi Jewish descent, exhibit a notably higher mutation rate [10–13]. According to Kuchenbaecker et al., female carriers of pathogenic *BRCA* variants face a lifetime breast cancer risk of approximately 70%. The ovarian cancer cumulative risk to age 80 years is considerably higher in women with a pathogenic *BRCA1* variant compared to those with a pathogenic *BRCA2* variant (*BRCA1*: 44%, *BRCA2*: 17%) [14]. However, recent studies indicate that the risks reported by Kuchenbaecker et al. may overestimate those for pathogenic *BRCA2* variant carriers: Current data show a cumulative breast cancer risk of 58% to age 80 years and the lifetime risk of ovarian cancer is also considered to be lower than previously assumed [15, 16]. Men with a pathogenic *BRCA1* variant exhibit a mildly elevated lifetime breast cancer risk of approximately 1%, while those with a pathogenic *BRCA2* variant have a lifetime risk of around 8% [17, 18]. Individuals with pathogenic *BRCA* variants face an elevated risk not only for breast and gynecologic cancers but also for pancreatic cancer (*BRCA1*: 1–3%, *BRCA2*: 3–5% by age 70), and male carriers for prostate cancer (*BRCA1*: 21%, *BRCA2*: 27% by age 75). Additionally, pathogenic *BRCA2* variants are associated with an increased risk of melanoma [19].

### Pathogenic *PALB2* variants

Besides pathogenic variants in the *BRCA* genes, alterations in other genes like *PALB2* (*FANCN*) can also contribute to HBOC [5]. In women carrying a pathogenic *PALB2* variant, the risk of developing breast cancer by age 80 is approximately 53%, while the risk for ovarian cancer stands at circa 5%. In comparison, male carriers have a considerably lower breast cancer risk, estimated at about 1% by age 80. Monoallelic pathogenic *PALB2* variants also increase the risk of pancreatic cancer (risk by age 80: 2% for women, 3% for men) [20]. Homozygous carriers of pathogenic *PALB2* variants develop Fanconi anemia [21].

### Further clinically relevant genes in the HBOC spectrum

A number of other hereditary tumor syndromes can be considered as a differential diagnosis to *PALB2*- and *BRCA*-associated HBOC. High-penetrance genes for breast cancer are *TP53* (Li-Fraumeni syndrome), *STK11* (Peutz-Jeghers syndrome), *PTEN* (Cowden syndrome, *PTEN* hamartoma tumor syndrome), and *CDH1* (Hereditary Diffuse Gastric Cancer) [19, 22]. The respective genes and their associated syndromes are discussed in detail in dedicated sections of this review.

Beyond the high-penetrance genes associated with breast cancer, moderate-risk genes, such as *ATM*, *BARD1*, and *CHEK2*, also contribute to hereditary breast cancer predisposition and are therefore considered part of the HBOC spectrum [22]. In addition to *BRCA1/2* and *PALB2*, ovarian cancer risk genes also include *RAD51C*, *RAD51D*, and *BRIP1*. However, pathogenic alterations in these genes are likely to cause only a modest increase in the risk of breast cancer [5, 19]. It is important to note that the genetic etiology remains unidentified in approximately 50% of clinical cases of HBOC [22].

#### Pathogenic *ATM* variants

Although certain pathogenic variants in the *ATM* gene, such as c.7271 T > G, are classified as “high-risk variants”, the *ATM* gene is generally regarded as a moderate-penetrance risk gene for breast cancer [19, 23, 24]. The heterozygote frequency of pathogenic variants in the general population is 0.4–2% [1, 23, 24]. A distinction is made between three different forms: heterozygotic/monoallelic, homozygotic/biallelic (classic ataxia telangiectasia), and non-classic form (attenuated phenotype) [25]. In contrast to ataxia telangiectasia, which would lead to severe neurological and pulmonary symptoms due to a biallelic loss-of-function, the monoallelic form is associated with an increased risk of breast cancer. The lifetime risk of developing breast cancer is estimated at 20–30% [22–24]. Particularly at a young age (under 50 years), the breast cancer risk is reported to be increased by up to fivefold [23]. In contrast, patients with a pathogenic *ATM* variant do not have an increased risk of developing contralateral breast cancer [26]. The data situation for other associated tumor diseases, such as prostate cancer or pancreatic cancer, is currently insufficient. The risk of developing ovarian cancer is, at most, only marginally increased [22, 27, 28].

#### Pathogenic *BARD1* variants

The *BARD1* gene was incorporated into the TruRisk® gene panel of the GC-HBOC in recent years as a moderate-risk gene for breast cancer [1]. *BARD1* stands for *BRCA1*-associated RING domain protein-1 and contributes to tumor suppression by promoting apoptosis and DNA double-strand repair [1, 29]. Carriers of pathogenic *BARD1* variants are assumed to have an at least twofold increased lifetime risk of breast cancer compared with the general population. However, reported risk estimates vary considerably across cohorts, with odds ratios reported between non-significant levels and up to 5.35 [22, 29–32]. Current evidence does not indicate an increased risk for gynecologic malignancies, particularly ovarian cancer [1, 22]. The role of pathogenic *BARD1* variants in other malignancies has not yet been fully elucidated, although some data suggest possible associations with tumors such as neuroblastoma [33].

#### Pathogenic *CHEK2* variants

The *CHEK2* gene encodes a serine/threonine kinase that plays a pivotal role in DNA damage response, cell cycle control, and apoptosis [34]. Germline pathogenic variants in *CHEK2* are associated with an increased susceptibility to malignancy. Depending on the specific variant, elevated cancer risks have been described for the breast, kidney, colon, thyroid, and, in men, the prostate [34–37]. Moreover, an association with non-Hodgkin lymphomas has been reported in the literature [34, 38]. The lifetime breast cancer risk conferred by *CHEK2* variants is generally considered moderate, corresponding to an approximately 1.5- to fourfold increase compared with the general population [35, 39]. It is influenced not only by the specific genetic alteration, but also by family history [35]. Conversely, no association has been established between *CHEK2* pathogenic variants and ovarian cancer risk [22].

#### Pathogenic *BRIP1* variants

*BRIP1* (*FANCJ*) encodes a helicase that interacts with *BRCA1* and contributes to DNA double-strand break repair [40]. While biallelic loss of function causes Fanconi anemia, heterozygous carriers of pathogenic *BRIP1* variants have been shown to face an increased susceptibility to ovarian cancer, with lifetime risks by age 80 estimated at approximately 4–9% [40, 41]. The onset of ovarian cancer in these carriers typically occurs later in life [41, 42]: In a large cohort, fewer than 10% of cases were diagnosed before age 50, about 30% between ages 50 and 59, and over 60% from 60 years of age onward [41]. Earlier studies proposed a twofold increase in breast cancer risk [43]. However, subsequent large-scale investigations have found no convincing evidence for a significant association [36, 42, 44]. Currently, no robust data are available regarding risks for other malignancies in pathogenic *BRIP1* variant carriers.

#### Pathogenic *RAD51C *and *RAD51D* variants

*RAD51C* (*FANCO*) is classified as a moderately penetrant breast cancer susceptibility gene. The estimated cumulative lifetime risk of breast cancer for heterozygous carriers of pathogenic *RAD51C* variants up to the age of 80 years ranges from approximately 15–30% [36, 45]. The individual risk may be higher in women with a family history of breast cancer. The estimated lifetime risk of ovarian cancer by age 80 years is reported to be 11%, and may similarly be elevated among individuals with a notable familial aggregation of ovarian malignancies [45]. Notably, the cumulative risk of ovarian cancer among women with pathogenic *RAD51C* variants was reported to be 1% by age 50, but to increase substantially thereafter, with a peak at 60–69 years of age [45]. At present, no robust evidence supports an association between pathogenic *RAD51C* variants and an increased risk for malignancies other than breast or ovarian cancer. As observed for other genes in the HBOC spectrum, biallelic pathogenic *RAD51C* variants are linked to chromosome instability disorders (Fanconi anemia-like syndrome) [1, 46].

Pathogenic variants in *RAD51D* confer comparable cancer risks, with a lifetime breast cancer risk of approximately 15–30% and an ovarian cancer risk of 13% by age 80 [36, 45]. Similar to pathogenic *RAD51C* variants, *RAD51D*-associated ovarian cancer risk remains low at younger ages (cumulative risk to age 50: 0.8%) but rises markedly thereafter. In contrast to *RAD51C*, however, the relative risk for carriers of pathogenic *RAD51D* variants reaches its peak earlier, typically between 50 and 59 years of age [45].

### Surveillance strategies and therapeutic implications in HBOC

In cases of HBOC, enhanced breast surveillance is recommended, with the specific scope and starting age varying by gene and national guideline [5, 22]. In general, European guidelines advocate for breast self-awareness and regular self-examinations starting at age 18. Additionally, regular clinical breast exams and mammography as well as annual MRI screenings are advised for a number of HBOC-associated pathogenic genetic variants. However, there are significant differences regarding the recommendations for breast sonography across Europe [5]. Recent ESMO (European Society for Medical Oncology) guidelines recommend sonography, especially for pathogenic *BRCA1* variant carriers < 40 years of age, between annual MRIs to ensure biannual imaging control [22]. In Germany, carriers of pathogenic variants in *ATM*, *BRCA1*, *BRCA2*, *BARD1*, *BRIP1*, *CDH1*, *CHEK2*, *PALB2*, *PTEN*, *RAD51C*, *RAD51D*, and *TP53* are eligible for the intensified breast cancer surveillance (IBCS) program, which includes annual breast MRI as a core component [1]. Additional imaging modalities, such as breast ultrasound and mammography, are offered depending on the underlying genetic alteration and age (e.g., mammography from 40 years onward). Surveillance protocols of the German IBCS are regularly updated to reflect evolving evidence. In general, female carriers of genetic risk variants may enter the program from the age of 30 and continue intensified surveillance until 70 years of age. For high-risk constellations, earlier initiation of IBCS is recommended, e.g., from age 20 in pathogenic *TP53* variant carriers and from age 25 in pathogenic *BRCA1/2* variant carriers [1, 47]. In this regard, the German approach differs from the ESMO guidelines, which advise starting intensified breast surveillance for women with pathogenic *BRCA1*/*2* variants at age 30 or 5 years before the youngest age of breast cancer onset in the family [22]. Risk-reducing surgery may be considered for females carrying pathogenic variants in high-risk genes such as *BRCA1/2* and — depending on family history — *TP53*, *PTEN*, *STK11*, *CDH1*, and *PALB2* [1, 22]. Before opting for risk-reducing mastectomy (RRM), a meticulous evaluation of the patient's age, comorbidities (including malignancies and their prognosis), anatomical characteristics, sexual health, and psychological well-being is imperative [22, 48].

Although intensified ovarian cancer screening lacks proven benefits, some national guidelines across Europe recommend regular transvaginal sonography and CA-125 tumor marker monitoring for high-risk groups [5, 22]. Risk-reducing salpingo-oophorectomy (RRSO) is the most effective method to reduce ovarian cancer risk. For *BRCA1*, surgery is usually recommended at 35–40 years, and for *BRCA2* at 40–45 years, after completion of childbearing. RRSO is also an option for carriers of pathogenic variants in *BRIP1*, *RAD51C*, and *RAD51D* (at 45–50 years) as well as *PALB2* (at the onset of menopause), depending on family history [1, 22].

In the context of HBOC, surveillance and prophylactic strategies are not limited to breast and ovarian malignancies. Given the elevated cancer susceptibility in other organ systems — depending on the specific germline pathogenic variant and detailed family history — comprehensive screening protocols are indicated. For instance, male pathogenic *BRCA2* variant carriers are advised to undergo prostate-specific antigen (PSA) testing starting at age 40, given their significantly increased risk for prostate cancer [22]. Furthermore, for individuals with a familial predisposition to pancreatic cancer and the presence of pathogenic variants in defined high-risk genes, yearly screening utilizing MRI-based imaging and/or endoscopic ultrasound is currently advocated to enable the early detection of pancreatic neoplasia [22, 49].

Given that HBOC can arise from a range of distinct genetic alterations, gene-specific characteristics and implications should be carefully considered in clinical decision-making. HBOC on the basis of a pathogenic *BRCA* variant not only influences cancer surveillance strategies but also has significant therapeutic implications, as the presence of such a genetic alteration constitutes a key criterion for the use of PARP (poly (ADP-ribose) polymerase) inhibitors across multiple tumor entities [49–52]. In carriers of pathogenic *ATM* variants, a possible increase in radiation sensitivity has been discussed, but there are no clear data available. Accordingly, radiotherapy should not be withheld after breast-conserving surgery for breast cancer, and affected individuals should receive standard-of-care treatment [53, 54]. In contrast, patients with ataxia telangiectasia appear to react more sensitively to radiotherapy and chemotherapy [55]. In the presence of a breast carcinoma, an individualized approach in the context of an interdisciplinary tumor conference is recommended for these special patients.

In summary, a comprehensive, multidisciplinary approach is necessary, giving the variety of cancers in various organ systems that are part of the HBOC.

## Li–Fraumeni syndrome (LFS)

Li-Fraumeni syndrome (LFS) is characterized by a genetic predisposition to a range of cancers, particularly in early childhood. It is caused by pathogenic variants in the *TP53* gene, which plays a pivotal role in regulating the cell cycle as a tumor suppressor, thereby significantly contributing to the prevention of tumor formation. As a result, carcinomas may manifest as early as childhood [56]. The most prevalent cancers observed in LFS patients up to the age of 15 years include adrenocortical carcinoma (ACC), choroid plexus carcinoma, rhabdomyosarcoma, and medulloblastoma. In later life, the following cancers predominantly occur: breast cancer, osteosarcoma, soft-tissue sarcoma (STS), leukemia, astrocytoma, glioblastoma, colorectal cancer (CRC), and lung cancer. Pancreatic cancer and prostate cancer are more characteristic of older adulthood in individuals with LFS [57]. LFS confers a lifetime cancer risk of about 70% in males and over 90% in females [57, 58]. Reported lifetime breast cancer risks for women with LFS range up to 80–90%, surpassing the risk linked to *BRCA1*- and *BRCA2*-related hereditary breast cancer [57, 59], whereas ESMO guidelines indicate a substantially lower lifetime risk of around 40% [22]. Due to the substantially elevated breast cancer risk, *TP53* has been included in the TruRisk^®^ gene panel of the GC-HBOC [1]. Besides, the modified Chompret criteria help identify individuals at increased risk for LFS who require genetic testing. In the context of breast cancer, the occurrence of early onset disease before age 31 or premenopausal breast cancer in combination with another LFS-associated malignancy in the patient or family history should raise suspicion for LFS [57, 60].

The rarity (estimated prevalence of germline *TP53* pathogenic variants in general population: 1:3,000 to 1:10,000) and heterogeneity of LFS have posed significant challenges in establishing a structured program for early detection and prevention [57, 61]. The involvement of affected individuals in specialized consultations or studies is highly recommended. In line with the ERN GENTURIS (European Reference Network for rare Genetic Tumor Risk Syndromes) guidelines, surveillance in individuals with LFS involves intensive, lifelong clinical and imaging-based assessments beginning in early childhood [62]. Clinical examinations are advised every six months in children and annually in adults. Whole-body MRI is recommended once per year from birth for carriers of especially high-cancer-risk *TP53* variants, defined as variants associated with childhood cancers in the index case or family, or those corresponding to dominant-negative missense variants, or in individuals with a prior history of chemotherapy or radiotherapy. All other individuals affected by pathogenic *TP53* variants are offered an annual whole-body MRI scan from the age of 18. In addition, brain MRI is recommended annually from birth in carriers of high-cancer-risk *TP53* variants and between ages 18 and 50 for all individuals with pathogenic *TP53* variants. Abdominal ultrasound is advised every six months from birth to 18 years [62]. According to the ERN GENTURIS guidelines, colonoscopy is reserved for those with prior abdominal radiotherapy or a strong family history suggestive of hereditary colorectal cancer predisposition [62]. By comparison, U.S. and Canadian guidelines similarly advocate comprehensive and age-adapted surveillance strategies, but differ in certain procedural details and timing: recommended screening protocols include frequent clinical examinations from birth (children: every 3–4 months, adults: every 6 months), annual full-body MRI as well as brain MRI from birth, and ultrasound of abdomen and pelvis in child- and adulthood, with intervals ranging from 3 to 12 months depending on age. Additional screening procedures encompass endoscopic evaluations, dermatologic assessments, and laboratory tests (e.g., serum PSA levels or differential blood counts), tailored to age and gender [63–65]. From a senological perspective, women with LFS are encouraged to perform breast self-examinations starting at age 18. Beginning at 20 years, a structured clinical breast examination every 6 months, alongside an annual breast MRI is recommended [62–65]. Recommendations regarding the use of mammography for individuals with LFS are included in U.S. and Canadian surveillance protocols, but they are not endorsed by the ERN GENTURIS guideline [62–64]. Affected females with LFS in Germany are eligible for inclusion in the IBCS program of the German Consortium for Familial Breast and Ovarian Cancer, starting at the age of 20 years [1]. The option of prophylactic bilateral mastectomy (risk-reducing bilateral mastectomy, RRBM) should be considered on an individual basis, taking into account the patient's family history and personal risk factors [1, 63, 65]. However, RRSO is not routinely recommended for patients with LFS [1, 65]. Structured early detection and cancer prevention are of critical importance for patients with LFS, as treatments such as radiation and conventional chemotherapy may elevate the risk of secondary cancers, which is higher than in the general population and, ideally, should be minimized. However, in cases where radiation or chemotherapy is clearly indicated, these treatments should proceed following a comprehensive review and interdisciplinary case discussion [65, 66].

New approaches in the management and early detection of LFS patients may include the development of screening methods based on cell-free DNA, such as liquid biopsy, for early and targeted cancer detection [67]. This would enable more selective and less frequent use of imaging studies, while concurrently enhancing patient safety. However, the standardization and implementation of such a program necessitate further research before it can be incorporated into clinical practice.

## Lynch syndrome

Lynch syndrome, also known as hereditary nonpolyposis colorectal cancer (HNPCC) syndrome, is an autosomal dominant inherited disorder characterized by germline pathogenic variants in DNA mismatch repair (MMR) genes *MLH1*, *MSH2*, *MSH6*, and/or *PMS2* [68]. However, pathogenic variants in the *EPCAM* gene with subsequent inactivation of *MSH2* are also known to be causative [68, 69]. These genetic alterations result in microsatellite instability (MSI), a hallmark molecular feature that predisposes affected individuals to early onset CRC and a spectrum of extracolonic malignancies, including endometrial, ovarian, gastric, small bowel, bile duct, pancreatic, urinary tract, and skin cancers as well as tumors of the central nervous system (CNS) [68, 70]. The risk profile for tumor development varies according to the specific mismatch repair gene involved [70]. Carriers of pathogenic variants in *MLH1* and *MSH2* exhibit the highest overall cancer risks among individuals with Lynch syndrome, with cumulative cancer incidences of approximately 81% (♀)/ 71% (♂) and 84% (♀)/ 75% (♂) at the age of 75 [70]. Compared to pathogenic variants in other MMR genes, *PMS2*-associated Lynch syndrome shows lower cancer susceptibility, with circa 10–13% lifetime risks for colorectal and endometrial cancer and relatively low incidence of other associated malignancies [70, 71].

Lynch syndrome, when considered from a gynecological perspective, is associated with elevated risks for endometrial and ovarian cancers in female carriers of pathogenic variants in MMR genes. Gene-specific cumulative risks for endometrial cancer by age 75 are estimated at 13% for *PMS2*, 37% for *MLH1*, 41% for *MSH6*, and 49% for *MSH2* [70]. Ovarian cancer risk also shows gene-dependent variation, ranging from approximately 3% in pathogenic *PMS2* variant carriers to 17% in pathogenic *MSH2* variant carriers [70]. It should be noted that these estimates are subject to wide confidence intervals, particularly for *PMS2*, which limits their statistical precision. The association between Lynch syndrome and breast cancer risk remains controversial. While certain studies suggest a link to pathogenic variants in MMR genes, current evidence is insufficient to confirm significant increase in breast cancer risk [32, 39, 70–74]. Owing to the lack of evidence-based data, no intensified surveillance for breast cancer is presently established for Lynch syndrome in Germany; accordingly, the standard national screening protocols are endorsed. To date, no screening method for the early detection of endometrial cancer in individuals with Lynch syndrome has been shown to improve survival, and current evidence is insufficient to support specific screening recommendations [75, 76]. Ovarian cancer likewise lacks effective and evidence-based early detection measures [76]. Risk-reducing hysterectomy with bilateral salpingo-oophorectomy should be discussed with female carriers following completion of childbearing, generally from age 40 onward, taking into account gene-specific cancer risks, family history, non-hereditary risk factors, patient age, and individual preferences [75–77]. Given the comparatively lower risk, consideration of risk-reducing adnexectomy in carriers of pathogenic *MSH6* or *PMS2* variants may appropriately be delayed until around the age of 50 [77].

In addition to gynecologic preventive care, patients with Lynch syndrome are advised to undergo regular colonoscopies, esophagogastroduodenoscopies, and — depending on family history — magnetic resonance cholangiopancreatography (MRCP) or endoscopic ultrasound for the early detection of pancreatic cancer [49, 77–79]. Aspirin chemoprevention has demonstrated promising results in clinical studies, although it is not yet part of widespread routine clinical practice [80].

## *DICER1* syndrome

*DICER1* syndrome is a rare hereditary condition arising from defective microRNA biogenesis, the precise population prevalence of which remains undetermined [81, 82]. Heterozygous carriers of pathogenic *DICER1* variants show a predisposition to a broad spectrum of both benign and malignant tumors, including but not limited to pleuropulmonary blastoma, neoplasms of the thyroid gland, and ovarian tumors (Sertoli–Leydig cell tumor, gynandroblastoma, and sarcoma) [81–83]. Tumor entities associated with pathogenic *DICER1* variants typically manifest at a young age (< 40 years) [83]. Aside from elevated neoplasia risk, clinical manifestations of *DICER1* syndrome comprise macrocephaly, renal and urinary tract anomalies, ocular defects, and distinctive dental morphology [83]. However, the phenotypic range has yet to be fully characterized [81]. Current recommendations for tumor surveillance in individuals carrying pathogenic *DICER1* variants entail regular clinical and imaging assessments, such as chest radiography or computed tomography (CT) and ultrasound of the thyroid, abdomen, and pelvis. Periodic ophthalmologic and neurological assessments are also advised [83]. Although experimental studies in cellular and animal models have explored potential therapeutic strategies, such as upregulation of *DICER1* expression through metformin or pharmacologic read-through of nonsense mutations, these approaches remain preliminary and have not yet been validated for clinical application in patients with *DICER1* syndrome [81].

## Hereditary Diffuse Gastric Cancer (HDGC)

Hereditary Diffuse Gastric Cancer (HDGC) is a rare autosomal dominant cancer predisposition syndrome (estimated incidence: 5–10 per 100 000 births worldwide) associated with an elevated risk of diffuse-type gastric cancer (DGC) and lobular breast carcinoma. The condition is predominantly driven by inactivating germline pathogenic variants in the *CDH1* (cadherin-1) tumor suppressor gene, while pathogenic variants in *CTNNA1* account for a smaller subset of cases [84]. Pathogenic variants in the *CDH1* gene are associated with a lifetime risk of gastric cancer by age 80 ranging from 33 to 70%, though substantial variability exists across studies. Men appear to be at higher risk. In contrast, the lifetime risk of breast cancer in affected women is estimated at 39–55%, with greater consistency observed among reported data [84–86]. To date, no robust evidence supports a substantially elevated risk for malignancies beyond gastric and breast cancer in individuals with germline pathogenic variants in *CDH1* [84].

The 2020 HDGC genetic testing guidelines recommend *CDH1* analysis in individuals or families who meet defined clinical criteria, which include either a family history of DGC and/or early onset lobular breast cancer, or individual features (e.g., early onset DGC; DGC in the context of Māori ancestry or cleft lip/palate; combined DGC and lobular breast cancer before age 70; bilateral early onset lobular breast cancer; gastric in situ signet ring cells/pagetoid spread of signet ring cells before age 50). If *CDH1* testing is negative despite fulfillment of these criteria, further analysis of *CTNNA1* should be considered [84]. It is important to note that in up to 70% of HDGC cases, the underlying genetic alteration has not yet been identified [87].

Carriers of germline pathogenic *CDH1* or *CTNNA1* variants are advised to undergo screening for *Helicobacter pylori* infection, with prompt initiation of eradication therapy in the event of a positive result [87]. In families with confirmed HDGC and a pathogenic *CDH1* or *CTNNA1* variant, prophylactic total gastrectomy should be offered from age 20. For those who decline or defer surgery, annual endoscopic surveillance by experienced specialists is recommended [84, 87]. In individuals with an HDGC-like phenotype without confirmed pathogenic *CDH1* or *CTNNA1* variants, yearly gastroduodenoscopy is advised for at least two years, with the option to extend the interval thereafter based on individualized risk assessment [84]. Similarly, in cases of hereditary lobular breast cancer associated with a pathogenic *CDH1* variant but without a family history of gastric cancer, endoscopic surveillance is advised annually [84, 87]. Breast surveillance in the context of hereditary diffuse gastric cancer and hereditary lobular breast cancer (HLBC) involves annual clinical examination and breast MRI beginning at age 30, complemented by annual mammography from age 40; consideration of risk-reducing mastectomy may be appropriate based on individual risk assessment [1, 84].

## Pathogenic *NBN* (*NBS1*) variants

Nijmegen breakage syndrome (NBS) is a rare autosomal recessive disorder characterized by chromosomal instability, immunodeficiency, and a high predisposition to malignancies, particularly lymphoid cancers [88]. The genetic basis of NBS lies in biallelic pathogenic variants in the *NBN* (also termed *NBS1)* gene, which encodes nibrin, a protein crucial for DNA double-strand break repair [88–90]. The most frequent pathogenic variant is c.657_661del5 [88]. Diagnosis relies on clinical findings, such as progressive microcephaly, dysmorphic facial features, immunodeficiency, and premature ovarian failure, confirmed by genetic testing [88]. Approximately 40% of NBS patients develop cancer by the age of 20 [88]. Owing to defective DNA repair mechanisms, individuals with NBS have reduced tolerance to chemotherapy and radiotherapy, presenting substantial challenges in oncologic management [89]. Hematopoietic stem cell transplantation has been shown to improve long-term survival in patients with lymphoid malignancies [91]. While the *NBN* (*NBS1*) gene is classically associated with autosomal recessive Nijmegen breakage syndrome (NBS), heterozygous carriers of pathogenic *NBN* variants may also have an elevated cancer risk. The 657del5 founder mutation, in particular, has been associated with a low-to-moderate increase in breast cancer risk in some studies, though findings have been inconsistent [39, 90, 92–94]. Currently, there are no established recommendations for enhanced breast cancer screening in individuals carrying monoallelic pathogenic *NBN* variants.

## Neurofibromatosis type 1 (NF1)

Neurofibromatosis type 1 (NF1) is an inherited autosomaldominant disorder caused by various heterogeneous alterations in the *NF1* gene, which encodes the protein neurofibromin and thus exerts a significant regulatory effect on the RAS signal pathway [95]. NF1 is characterized by distinctive cutaneous manifestations, such as café-au-lait macules, inguinal/axillary freckling, and neurofibromas (benign tumors of the peripheral nervous system), as well as a predisposition to the development of malignant neoplasms. Compared to the general population, the tumor risk is 2.7 times higher, with particular emphasis on malignant peripheral nerve sheath tumors (MPNST), gliomas, leukemia, gastrointestinal stromal tumors, pheochromocytomas, and rhabdomyosarcomas [95–97]. Moreover, carriers of pathogenic *NF1* variants exhibit a significantly increased risk of breast cancer in early to mid-adulthood, particularly between ages 30 and 50, whereas a possible elevation in cumulative lifetime risk, estimated at about twofold compared with the general population, remains uncertain [36, 98, 99]. Cumulative risk for contralateral breast cancer risk is reported to be elevated in pathogenic *NF1* variant carriers, estimated at 26.5% over 20 years [100]. Additionally, prognosis tends to be poorer due to later-stage diagnoses and more aggressive tumor biology [98, 100]. Interestingly, somatic pathogenic *NF1* variants may contribute to endocrine resistance in advanced breast cancer; however, their precise clinical relevance in individuals with germline pathogenic *NF1* variants remains to be fully elucidated [101]. Due to the broad tumor spectrum associated with NF1, affected individuals are offered receive tailored surveillance, ideally coordinated within specialized centers with NF1 expertise. In line with the current ERN GENTURIS guidelines, individuals with NF1 are recommended to undergo annual breast MRI screening between the ages of 30 and 50. From age 51 onward, standard breast cancer screening protocols apply [102]. RRBM should not be routinely considered for women with NF1 unless additional risk factors, such as a significant family history, justify reclassification into a high-risk category. For other gynecologic malignancies, enhanced surveillance beyond standard recommendations is generally not indicated in the context of NF1 [102].

## Peutz-Jeghers syndrome (PJS)

Peutz-Jeghers syndrome (PJS) is an autosomal dominant cancer predisposition syndrome, with key clinical features including mucocutaneous pigmentation and gastrointestinal polyposis [103]. In 30–80% of cases, a pathogenic germline variant in the serine/threonine kinase 11 *STK11* (*LKB1*) gene, located on chromosome 19p13.3, can be identified [103]. PJS patients are estimated to carry lifetime malignancy risk of up to 80–85% by the age of 70 [103, 104]. Gastrointestinal malignancies are particularly prevalent, with cumulative risks estimated at 29% for gastric, 13% for small bowel, and 39% for colorectal cancer. The likelihood of developing pancreatic cancer lies between 11 and 36%, while the risk of lung cancer is estimated at 7–17%. The cumulative risk of testicular cancer in affected males is approximately 9%. Female PJS patients face significantly elevated risks of breast cancer (32–54%), as well as gynecologic cancers, including malignancies of the uterine corpus and cervix (each approximately 10%) and ovarian tumors, with risks reaching up to 21% [105]. In PJS, beyond the potential for ovarian carcinoma, mucinous ovarian neoplasms and sex cord-stromal tumors with annular tubules are particularly characteristic findings [103–105]. Given the broad tumor spectrum and elevated cancer risks, individuals with PJS are advised to undergo intensive, multi-organ cancer surveillance, although the optimal surveillance protocol remains a subject of ongoing debate. For breast cancer screening in females, the EHTG (European Hereditary Tumor Group) guideline suggests self-examination from age 18, accompanied by clinical breast exams every 6–12 months and annual breast MRI starting at age 25 as well as annual mammography earliest from the age of 30. RRBM in the context of PJS should be considered on an individual basis, given the limited data regarding its clinical benefit [1, 106]. Owing to the increased risk of gynecologic cancers in PJS, early comprehensive counseling on cancer risk, red flag symptoms, and reproductive implications is essential. Annual gynecologic assessments with cervical screening [cytology and human papillomavirus (HPV) testing] are recommended, with attention to the higher incidence of cervical adenocarcinoma. Regular transvaginal ultrasound is further advised in line with EHTG guidelines [106]. As part of gastrointestinal management in PJS, baseline oesophago-gastroduodenoscopy and colonoscopy are indicated at the age of 8 years. If polyps are detected, follow-up endoscopic evaluations are warranted every 1–3 years, depending on polyp burden. In the absence of findings, routine oesophago-gastroduodenoscopy and colonoscopy are offered from the age of 18. For small-bowel surveillance, video capsule endoscopy or MRI are available modalities, with baseline assessment also advised at age 8, followed by repeated evaluations every 1–3 years, based on phenotype. If gastrointestinal polyps are present, surgical intervention may be recommended depending on polyp size and clinical symptoms [106]. In Germany, pancreatic cancer screening using MRI-based imaging and/or endoscopic ultrasound is available for individuals carrying a pathogenic germline *STK11* variants [49].

## *PTEN* hamartoma tumor syndrome (PHTS)

The *PTEN* hamartoma tumor syndrome (PHTS) spectrum encompasses distinct clinical entities, including Cowden syndrome (CS) and Bannayan–Riley–Ruvalcaba syndrome (BRRS) [107, 108].

CS, the most common clinical manifestation of PHTS, is a rare (estimated prevalence 1:200,000) autosomal dominant disorder characterized by germline pathogenic variants in the *PTEN* (phosphatase and tensin homolog) tumor suppressor gene located on chromosome 10q23.3. Interestingly, while primarily inherited, up to 11–48% of cases result from de novo mutations [108, 109]. The *PTEN* gene encodes a phosphatase essential for downregulating the PI3K/AKT/mTOR (PI3K: phosphatidylinositol 3-kinase, mTOR: mammalian target of rapamycin) signaling pathway, and thus for controlling cell survival and proliferation [107, 109]. Pathogenic *PTEN* variants promote tumorigenesis [107, 109, 110]. The clinical presentation of CS is highly variable, with manifestations ranging from benign hamartomas to malignant tumors affecting multiple organ systems. Breast cancer is the most frequent malignancy in women with CS, with a reported lifetime risk ranging from 25 to 50% [108, 110–112]. Emerging data, however suggest substantially higher risks, estimating lifetime probabilities up to 85% [108, 110, 112–115]. Accordingly, the exact magnitude of lifetime breast cancer risk remains a subject of ongoing discussion in the literature [108, 110, 112–114]. Other malignancies commonly associated with CS comprise endometrial cancer (2–28%), thyroid cancer (3–38%, particularly follicular and papillary carcinoma), renal cell carcinoma (up to 34%) colorectal cancer (9–20%), and melanoma (0–6%) [107, 108, 110, 115]. In the context of CS, cancer often presents at a younger age, with breast cancer typically occurring in the fourthtofifth decade of life and endometrial cancer presenting at a median age of approximately 48 years, for example [107, 110]. Typical benign findings in CS include macrocephaly, mucocutaneous lesions, such as trichilemmomas, oral papillomas, acral keratoses, and a wide variety of gastrointestinal polyps, including hamartomatous, hyperplastic, adenomatous, and ganglioneuromatous polyps [112, 116, 117]. A diagnosis of CS is based on criteria established by the International Cowden Consortium (ICC) and further refined by the National Comprehensive Cancer Network (NCCN), which list breast and endometrial cancer, among others, as major criteria [112, 117].

Approximately 80% of individuals meeting the clinical criteria for Cowden syndrome have detectable germline pathogenic *PTEN* variants, although this rate varies between 30 and 85% depending on the diagnostic criteria and testing methods used [117, 118]. Besides *PTEN*, other genetic aberrations have been implicated in Cowden syndrome and its related conditions within the PHTS spectrum. Pathogenic variants in the *SDHB*, *SDHC*, and *SDHD* genes, commonly associated with hereditary paraganglioma and pheochromocytoma syndromes, have been identified in individuals with CS-like features [110].

The management of CS necessitates a multidisciplinary approach, with a primary focus on cancer screening and risk reduction strategies. Surveillance protocols recommended by ERN GENTURIS include annual thyroid ultrasound from age 18, renal ultrasound every two years starting at age 40, baseline colonoscopy between ages 35 and 40 years with follow-up intervals based on polyp burden, and a dermatologic assessment at age 30 [115]. Furthermore, women are offered annual breast MRI starting at age 30, complemented by mammography every two years from age 40 as part of an intensified breast cancer screening [115]. While international guidelines are broadly aligned, subtle differences persist, particularly with respect to the recommended age of initiation and screening intervals [117, 119]. The NCCN and Japanese practice guidelines further advocate breast-self-examination from age 18 and advise clinical breast examinations starting at age 25 [117, 119]. Although annual transvaginal ultrasound or endometrial biopsy from age 30 has been proposed for early endometrial cancer detection in CS, no screening method has yet been shown to improve survival, and current data remain inadequate to support definitive guidelines [75, 115, 117]. Risk-reducing hysterectomy should be discussed with female patients following completion of childbearing, taking into account family history, non-hereditary risk factors, patient age, and individual preferences [75, 119]. To date, there is no evidence that RRBM improves overall survival in women with CS [117]. While — according to ERN GENTURIS guidelines — RRBM should be offered, the potential benefit of prophylactic breast surgery should be carefully evaluated on a case-by-case basis [1, 115, 117].

BRRS is another clinical entity within the PHTS spectrum, caused by germline pathogenic variants in the *PTEN* gene. Characteristic clinical features include macrocephaly, developmental delay, autism spectrum disorders, pigmented penile macules, mucocutaneous lesions, thyroid abnormalities, lipomas, gastrointestinal polyps, and vascular malformations. Due to the rarity of the condition, available data are largely limited to case reports and small case series. While an increased cancer risk is presumed — similar to other *PTEN*-related disorders — current evidence remains insufficient to establish standardized surveillance protocols [120].

## Pathogenic *SMARCA4* variants

The *SMARCA4* gene encodes the ATP-dependent chromatin remodeling protein SMARCA4 [121]. Carriers of pathogenic *SMARCA4* variants face an elevated cancer risk, in the form of rhabdoid tumor predisposition syndrome type 2 [121, 122]. The spectrum of associated malignancies includes, but is not limited to SMARCA4-deficient undifferentiated uterine sarcoma as well as the small cell carcinoma of the ovary, hypercalcemic type (SCCOHT), a rare and highly aggressive ovarian neoplasm, typically arising in premenopausal women [121–126]. Due to the rarity of germline pathogenic *SMARCA4* variants, standardized recommendations for intensified cancer surveillance and preventive strategies are lacking [127]. Regular clinical and imaging assessments from early childhood have been proposed in the literature [127]. RSSO may be considered after completion of childbearing in the context of family history [127]. However, this option should be considered with caution, as supporting evidence remains limited, and carefully weighed against potential risks.

## Tuberous sclerosis

Tuberous sclerosis complex (TSC) is an autosomal dominant inherited disorder (incidence: 1: 6,000–1:10,000 live births) resulting from pathogenic variants in *TSC1* or *TSC2* and leading to hamartomatous lesions in various organs [128, 129]. Central to the pathophysiology of TSC is a dysregulation of mTOR signaling, while it is assumed that other proteins and signaling pathways may also play a contributory role [128]. Clinical presentation can differ widely and there is no consistent genotype–phenotype correlation [128]. However, genetic alterations in *TSC2* typically result in higher disease severity than pathogenic *TSC1* variants [128]. Characteristic manifestations of TSC comprise cutaneous lesions, neurological manifestations, and tumor formation [128, 129]. The tumor spectrum of TSC includes perivascular epithelioid cell tumors (PEComas), with several reports in the literature documenting uterine manifestations of these lesions in TSC patients [130–132]. PEComas represent a rare pathological entity that is predominantly benign, although malignant forms have been reported [130, 133]. Clinical symptoms are often non-specific. Affected individuals may, however, present with irregular vaginal bleeding or abdominal pain [132, 133]. Although the use of mTOR inhibitors in the management of TSC-associated tumors is well established, and initial studies have demonstrated their potential efficacy in PEComas, surgical intervention currently constitutes the central component of therapy for uterine PEComas [129, 132, 133]. The optimal management of uterine PEComas remains a matter of ongoing scientific debate, and further research is required to establish evidence-based therapeutic strategies [132]. Current surveillance recommendations for TSC patients encompass neurological assessments including electroencephalography, dermatologic, ophthalmologic, and dental evaluations, cardiac diagnostics such as electrocardiography and echocardiography, as well as imaging studies of the CNS, kidneys, and lungs [128, 129]. A dedicated TSC-specific gynecologic screening protocol has not been established to date [129]. Gynecologic evaluation is performed on an individualized basis in the presence of clinical symptoms.

## Conclusion

The landscape of hereditary cancer predisposition in senologic and gynecologic oncology extends far beyond *BRCA1/2*-associated HBOC. Genetic tumor syndromes, such as Li-Fraumeni syndrome, Lynch syndrome, *DICER1* syndrome, Hereditary Diffuse Gastric Cancer, Neurofibromatosis type 1, Peutz-Jeghers syndrome, *PTEN* hamartoma tumor syndrome, Tuberous Sclerosis, and pathogenic variants in *NBN* and *SMARCA4,* illustrate the genetic and phenotypic heterogeneity underlying inherited breast and gynecologic malignancies.

A comprehensive understanding of these entities is essential for integrating hereditary risk assessment in women’s cancer into oncologic practice. Awareness of their characteristic clinical constellations facilitates the timely initiation of genetic diagnostics, which in turn enables risk-adapted prevention, individualized surveillance, and precision therapeutics. The spectrum of preventive measures ranges from clinical and imaging-based assessments to risk-reducing surgery.

As the catalog of clinically relevant genetic alterations continues to expand, the management of hereditary tumor syndromes will grow in complexity and increasingly rely on multidisciplinary expertise and structured care pathways within specialized centers.

## Data Availability

No datasets were generated or analysed during the current study.

## Abbreviations

ACC: Adrenocortical carcinoma
*BARD1*: *BRCA1*-associated RING domain protein-1
BRRS: Bannayan–Riley–Ruvalcaba syndrome
CDH1: Cadherin-1
CNS: Central nervous system
CPC: Choroid plexus carcinoma
CRC: Colorectal cancer
CS: Cowden syndrome
CT: Computed tomography
DGC: Diffuse-type gastric cancer
EHTG: European Hereditary Tumor Group
ERN GENTURIS: European Reference Network for rare Genetic Tumor Risk Syndromes
ESMO: European Society for Medical Oncology
GC-HBOC: German Consortium for Familial Breast and Ovarian Cancer
HBOC: Hereditary Breast and Ovarian Cancer syndrome
HDGC: Hereditary Diffuse Gastric Cancer
HNPCC: Hereditary nonpolyposis colorectal cancer
HPV: Human papillomavirus
IBCS: Intensified breast cancer surveillance
ICC: International Cowden Consortium
LFS: Li–Fraumeni syndrome
MMR: Mismatch repair
MPNST: Malignant peripheral nerve sheath tumors
MRCP: Magnetic resonance cholangiopancreatography
MRI: Magnetic resonance imaging
MSI: Microsatellite instability
mTOR: Mammalian target of rapamycin
NBS: Nijmegen breakage syndrome
NCCN: National Comprehensive Cancer Network
NF1: Neurofibromatosis type 1
PARP: Poly (ADP-ribose) polymerase
PEComas: Perivascular epithelioid cell tumors
PHTS: *PTEN* Hamartoma tumor syndrome
PI3K: Phosphatidylinositol 3-kinase
PJS: Peutz-Jeghers syndrome
PSA: Prostate-specific antigen
*PTEN*: Phosphatase and tensin homolog
RRBM: Risk-reducing bilateral mastectomy
RRM: Risk-reducing mastectomy
RRSO: Risk-reducing salpingo-oophorectomy
SCCOHT: Small cell carcinoma of the ovary, hypercalcemic type
*STK11*: Serine/threonine kinase 11
STS: Soft tissue sarcoma
TSC: Tuberous sclerosis complex

## References

[1] Rhiem K, Auber B, Briest S et al (2022) Consensus recommendations of the German consortium for hereditary breast and ovarian cancer. Breast Care 17:199–207 35702495 10.1159/000516376 PMC9149395

[2] Nakamura S, Kwong A, Kim SW et al (2016) Current status of the management of hereditary breast and ovarian cancer in Asia: first report by the Asian BRCA Consortium. Public Health Genomics 19(1):53–60. 10.1159/000441714 26575363 10.1159/000441714

[3] Kamga KK, Marlyse PF, Nguefack S et al (2025) Advancing genetic services in African healthcare: challenges, opportunities, and strategic insights from a scoping review. Hum Genet Genomics Adv 6:100439. 10.1016/j.xhgg.2025.100439 10.1016/j.xhgg.2025.100439 PMC12041751 40211537

[4] Yoshida R (2021) Hereditary breast and ovarian cancer (HBOC): review of its molecular characteristics, screening, treatment, and prognosis. Breast Cancer 28:1167–1180 32862296 10.1007/s12282-020-01148-2 PMC8514387

[5] Marmolejo DH, Wong MYZ, Bajalica-Lagercrantz S et al (2021) Overview of hereditary breast and ovarian cancer (HBOC) guidelines across Europe. Eur J Med Genet 64:104350 34606975 10.1016/j.ejmg.2021.104350

[6] Lux MP, Bani MR, Fasching PA, et al. (2016) Hereditary breast and ovarian cancer. Mol Basis Hum Cancer: 401–421

[7] Gupta S, Rajappa S, Advani S et al (2021) Prevalence of BRCA1 and BRCA2 mutations among patients with ovarian, primary peritoneal, and fallopian tube cancer in India: a multicenter cross-sectional study. JCO Glob Oncol 7:849–861 34101484 10.1200/GO.21.00051 PMC8457852

[8] Harter P, Hauke J, Heitz F et al (2017) Prevalence of deleterious germline variants in risk genes including BRCA1/2 in consecutive ovarian cancer patients (AGO-TR-1). PLoS ONE 12:e0186043 29053726 10.1371/journal.pone.0186043 PMC5650145

[9] Kennedy RD, D’Andrea AD (2005) The Fanconi anemia/BRCA pathway: new faces in the crowd. Genes Dev 19:2925–2940. 10.1101/gad.1370505 16357213 10.1101/gad.1370505

[10] Warner E, Foulkes W, Goodwin P et al (1999) Prevalence and penetrance of BRCA1 and BRCA2 gene mutations in unselected Ashkenazi Jewish women with breast cancer. J Natl Cancer Inst 91:1241–1247 10413426 10.1093/jnci/91.14.1241

[11] Roa BB, Boyd AA, Volcik K et al (1996) Ashkenazi Jewish population frequencies for common mutations in BRCA1 and BRCA2. Nat Genet 14:185–187 8841191 10.1038/ng1096-185

[12] Struewing JP, Hartge P, Wacholder S et al (1997) The risk of cancer associated with specific mutations of BRCA1 and BRCA2 among Ashkenazi Jews. N Engl J Med 336:1401–1408 9145676 10.1056/NEJM199705153362001

[13] Nelson HD, Pappas M, Cantor A et al (2019) Risk assessment, genetic counseling, and genetic testing for BRCA-related cancer in women: updated evidence report and systematic review for the US Preventive Services Task Force. JAMA 322:666–685 31429902 10.1001/jama.2019.8430

[14] Kuchenbaecker KB, Hopper JL, Barnes DR et al (2017) Risks of breast, ovarian, and contralateral breast cancer for BRCA1 and BRCA2 mutation carriers. JAMA 317:2402–2416 28632866 10.1001/jama.2017.7112

[15] Ficorella L, Yang X, Easton DF et al (2024) BOADICEA model: updates to the BRCA2 breast cancer risks for ages 60 years and older. BJC Rep 2:53 39072245 10.1038/s44276-024-00079-1 PMC11269170

[16] Ficorella L, Yang X, Mavaddat N et al (2025) Adapting the BOADICEA breast and ovarian cancer risk models for the ethnically diverse UK population: Epidemiology. Br J Cancer 2025:1–12 10.1038/s41416-025-03117-y PMC12449465 40676226

[17] Tai YC, Domchek S, Parmigiani G et al (2007) Breast cancer risk among male BRCA1 and BRCA2 mutation carriers. J Natl Cancer Inst 99:1811–1814 18042939 10.1093/jnci/djm203 PMC2267289

[18] Evans D, Susnerwala I, Dawson J et al (2010) Risk of breast cancer in male BRCA2 carriers. J Med Genet 47:710–711 20587410 10.1136/jmg.2009.075176

[19] Petrucelli N, Daly MB and Pal T (2022) BRCA1-and BRCA2-associated hereditary breast and ovarian cancer 20301425

[20] Yang X, Leslie G, Doroszuk A et al (2020) Cancer risks associated with germline PALB2 pathogenic variants: an international study of 524 families. J Clin Oncol 38:674–685 31841383 10.1200/JCO.19.01907 PMC7049229

[21] Ducy M, Sesma-Sanz L, Guitton-Sert L et al (2019) The tumor suppressor PALB2: inside out. Trends Biochem Sci 44:226–240. 10.1016/j.tibs.2018.10.008 30638972 10.1016/j.tibs.2018.10.008

[22] Sessa C, Balmaña J, Bober S et al (2023) Risk reduction and screening of cancer in hereditary breast-ovarian cancer syndromes: ESMO clinical practice guideline☆. Ann Oncol 34:33–47 36307055 10.1016/j.annonc.2022.10.004

[23] Thompson D, Duedal S, Kirner J et al (2005) Cancer risks and mortality in heterozygous ATM mutation carriers. J Natl Cancer Inst 97:813–822 15928302 10.1093/jnci/dji141

[24] Jerzak K, Mancuso T, Eisen A (2018) Ataxia–telangiectasia gene (ATM) mutation heterozygosity in breast cancer: a narrative review. Curr Oncol 25:e176 29719442 10.3747/co.25.3707 PMC5927797

[25] Garutti M, Foffano L, Mazzeo R et al (2023) Hereditary cancer syndromes: a comprehensive review with a visual tool. Genes 14:1025 37239385 10.3390/genes14051025 PMC10218093

[26] Yadav S, Boddicker NJ, Na J et al (2023) Contralateral breast cancer risk among carriers of germline pathogenic variants in ATM, BRCA1, BRCA2, CHEK2, and PALB2. J Clin Oncol 41:1703–1713 36623243 10.1200/JCO.22.01239 PMC10022863

[27] Bernards SS, Norquist BM, Harrell MI et al (2016) Genetic characterization of early onset ovarian carcinoma. Gynecol Oncol 140:221–225 26718727 10.1016/j.ygyno.2015.12.017 PMC4869974

[28] Kurian AW, Hughes E, Handorf EA et al (2017) Breast and ovarian cancer penetrance estimates derived from germline multiple-gene sequencing results in women. JCO Precis Oncol 1:1–12 35172496 10.1200/PO.16.00066

[29] Weber-Lassalle N, Borde J, Weber-Lassalle K et al (2019) Germline loss-of-function variants in the BARD1 gene are associated with early-onset familial breast cancer but not ovarian cancer. Breast Cancer Res 21(1):55. 10.1186/s13058-019-1137-9 31036035 10.1186/s13058-019-1137-9 PMC6489184

[30] Slavin TP, Maxwell KN, Lilyquist J et al (2017) The contribution of pathogenic variants in breast cancer susceptibility genes to familial breast cancer risk. NPJ Breast Cancer 3(1):22. 10.1038/s41523-017-0024-8 28649662 10.1038/s41523-017-0024-8 PMC5466608

[31] Couch FJ, Shimelis H, Hu C et al (2017) Associations between cancer predisposition testing panel genes and breast cancer. JAMA Oncol 3:1190–1196. 10.1001/jamaoncol.2017.0424 28418444 10.1001/jamaoncol.2017.0424 PMC5599323

[32] Lu H-M, Li S, Black MH et al (2019) Association of breast and ovarian cancers with predisposition genes identified by large-scale sequencing. JAMA Oncol 5:51–57 30128536 10.1001/jamaoncol.2018.2956 PMC6439764

[33] Kim J, Vaksman Z, Egolf LE et al (2024) Germline pathogenic variants in neuroblastoma patients are enriched in BARD1 and predict worse survival. JNCI J Natl Cancer Inst 116:149–159 37688579 10.1093/jnci/djad183 PMC10777667

[34] Apostolou P, Papasotiriou I (2017) Current perspectives on CHEK2 mutations in breast cancer. Breast Cancer (Dove Med Press) 9:331–335. 10.2147/BCTT.S111394. (**20170512**) 28553140 10.2147/BCTT.S111394 PMC5439543

[35] Cybulski C, Wokolorczyk D, Jakubowska A et al (2011) Risk of breast cancer in women with a CHEK2 mutation with and without a family history of breast cancer. J Clin Oncol 29(3747–3752):20110829. 10.1200/JCO.2010.34.0778 10.1200/JCO.2010.34.0778 21876083

[36] Breast Cancer Association C, Dorling L, Carvalho S et al (2021) Breast Cancer risk genes—association analysis in more than 113,000 women. N Engl J Med 384:428–439. 10.1056/NEJMoa1913948 33471991 10.1056/NEJMoa1913948 PMC7611105

[37] Cybulski C, Gorski B, Huzarski T et al (2004) CHEK2 is a multiorgan cancer susceptibility gene. Am J Hum Genet 75:1131–1135. 10.1086/426403. (**20041018**) 15492928 10.1086/426403 PMC1182149

[38] Havranek O, Kleiblova P, Hojny J et al (2015) Association of germline CHEK2 gene variants with risk and prognosis of non-Hodgkin lymphoma. PLoS ONE 10(10):e0140819. 10.1371/journal.pone.0140819 26506619 10.1371/journal.pone.0140819 PMC4624763

[39] Couch FJ, Shimelis H, Hu C et al (2017) Associations between cancer predisposition testing panel genes and breast cancer. JAMA Oncol 3:1190–1196 28418444 10.1001/jamaoncol.2017.0424 PMC5599323

[40] Rafnar T, Gudbjartsson DF, Sulem P et al (2011) Mutations in BRIP1 confer high risk of ovarian cancer. Nat Genet 43(11):1104–1107. 10.1038/ng.955 21964575 10.1038/ng.955

[41] Ramus SJ, Song H, Dicks E et al (2015) Germline mutations in the BRIP1, BARD1, PALB2, and NBN genes in women with ovarian cancer. J Natl Cancer Inst. 107:20150827. 10.1093/jnci/djv214 10.1093/jnci/djv214 PMC4643629 26315354

[42] Weber-Lassalle N, Hauke J, Ramser J et al (2018) BRIP1 loss-of-function mutations confer high risk for familial ovarian cancer, but not familial breast cancer. Breast Cancer Res 20(1):7. 10.1186/s13058-018-0935-9 29368626 10.1186/s13058-018-0935-9 PMC5784717

[43] Seal S, Thompson D, Renwick A et al (2006) Truncating mutations in the Fanconi anemia J gene BRIP1 are low-penetrance breast cancer susceptibility alleles. Nat Genet 38(1239–1241):20061008. 10.1038/ng1902 10.1038/ng1902 17033622

[44] Easton DF, Lesueur F, Decker B et al (2016) No evidence that protein truncating variants in BRIP1 are associated with breast cancer risk: implications for gene panel testing. J Med Genet 53:298–309. 10.1136/jmedgenet-2015-103529. (**20160226**) 26921362 10.1136/jmedgenet-2015-103529 PMC4938802

[45] Yang X, Song H, Leslie G et al (2020) Ovarian and breast cancer risks associated with pathogenic variants in RAD51C and RAD51D. J Natl Cancer Inst 112:1242–1250. 10.1093/jnci/djaa030 32107557 10.1093/jnci/djaa030 PMC7735771

[46] Somyajit K, Subramanya S, Nagaraju G (2010) RAD51C: a novel cancer susceptibility gene is linked to Fanconi anemia and breast cancer. Carcinogenesis 31:2031–2038. 10.1093/carcin/bgq210 20952512 10.1093/carcin/bgq210 PMC2994284

[47] Bick U, Engel C, Krug B et al (2019) High-risk breast cancer surveillance with MRI: 10-year experience from the German consortium for hereditary breast and ovarian cancer. Breast Cancer Res Treat 175:217–228 30725383 10.1007/s10549-019-05152-9

[48] Tauber N, Amann N, Dannehl D et al (2025) Therapy of early breast cancer: current status and perspectives. Arch Gynecol Obstet. 10.1007/s00404-025-08028-0 40261372 10.1007/s00404-025-08028-0 PMC12334469

[49] Leitlinienprogramm Onkologie (Deutsche Krebsgesellschaft DK, AWMF) (2024) S3-Leitlinie Exokrines Pankreaskarzinom, Langversion 3.1

[50] Cortesi L, Rugo HS, Jackisch C (2021) An overview of PARP inhibitors for the treatment of breast cancer. Target Oncol 16:255–282 33710534 10.1007/s11523-021-00796-4 PMC8105250

[51] Teyssonneau D, Margot H, Cabart M et al (2021) Prostate cancer and PARP inhibitors: progress and challenges. J Hematol Oncol 14:51 33781305 10.1186/s13045-021-01061-x PMC8008655

[52] Lux MP, Fasching PA (2023) Breast cancer and genetic BRCA1/2 testing in routine clinical practice: why, when and for whom? Geburtshilfe Frauenheilkd 83:310–320 36908286 10.1055/a-1929-2629 PMC9998182

[53] Reiner AS, Robson ME, Mellemkjær L et al (2020) Radiation treatment, ATM, BRCA1/2, and CHEK2* 1100delC pathogenic variants and risk of contralateral breast cancer. JNCI J Natl Cancer Inst 112:1275–1279 32119081 10.1093/jnci/djaa031 PMC7735763

[54] McDuff SG, Bellon JR, Shannon KM et al (2021) ATM variants in breast cancer: implications for breast radiation therapy treatment recommendations. Int J Radiat Oncol Biol Phys 110:1373–1382 33545302 10.1016/j.ijrobp.2021.01.045

[55] Bernstein JL, Haile RW, Stovall M et al (2010) Radiation exposure, the ATM Gene, and contralateral breast cancer in the women’s environmental cancer and radiation epidemiology study. J Natl Cancer Inst 102:475–483 20305132 10.1093/jnci/djq055 PMC2902825

[56] Kratz CP, Steinke-Lange V, Spier I et al (2022) Overview of the clinical features of Li–Fraumeni syndrome and the current European ERN GENTURIS guideline. Geburtshilfe Frauenheilkd 82:42–49 35027859 10.1055/a-1541-7912 PMC8747895

[57] Schneider K ZK, Nichols KE, Levine AS, Garber J (1999) Li-fraumeni syndrome. In: GeneReviews®[Internet]. University of Washington, Seattle 1993–2025. Seattle (WA), [Updated 2025 May 1] 20301488

[58] Fortuno C, Feng B-J, Carroll C et al (2024) Cancer risks associated with TP53 pathogenic variants: maximum likelihood analysis of extended pedigrees for diagnosis of first cancers beyond the Li–Fraumeni syndrome spectrum. JCO Precis Oncol 8:e2300453 38412388 10.1200/PO.23.00453 PMC10914239

[59] Schon K, Tischkowitz M (2018) Clinical implications of germline mutations in breast cancer: TP53. Breast Cancer Res Treat 167:417–423 29039119 10.1007/s10549-017-4531-y PMC5790840

[60] Bougeard G, Renaux-Petel M, Flaman J-M et al (2015) Revisiting Li–Fraumeni syndrome from TP53 mutation carriers. J Clin Oncol 33:2345–2352 26014290 10.1200/JCO.2014.59.5728

[61] Gargallo P, Yáñez Y, Segura V et al (2020) Li–Fraumeni syndrome heterogeneity. Clin Transl Oncol 22:978–988 31691207 10.1007/s12094-019-02236-2

[62] Frebourg T, Bajalica Lagercrantz S, Oliveira C et al (2020) Guidelines for the Li–Fraumeni and heritable TP53-related cancer syndromes. Eur J Hum Genet 28:1379–1386. 10.1038/s41431-020-0638-4 32457520 10.1038/s41431-020-0638-4 PMC7609280

[63] Kratz CP, Achatz MI, Brugieres L et al (2017) Cancer screening recommendations for individuals with Li–Fraumeni syndrome. Clin Cancer Res 23:e38–e45 28572266 10.1158/1078-0432.CCR-17-0408

[64] Villani A, Shore A, Wasserman JD et al (2016) Biochemical and imaging surveillance in germline TP53 mutation carriers with Li–Fraumeni syndrome: 11 year follow-up of a prospective observational study. Lancet Oncol 17:1295–1305 27501770 10.1016/S1470-2045(16)30249-2

[65] Achatz MI, Villani A, Bertuch AA et al (2025) Update on cancer screening recommendations for individuals with Li–Fraumeni syndrome. Clin Cancer Res 31:1831–1840 40072304 10.1158/1078-0432.CCR-24-3301

[66] Hendrickson PG, Luo Y, Kohlmann W et al (2020) Radiation therapy and secondary malignancy in Li-Fraumeni syndrome: a hereditary cancer registry study. Cancer Med 9:7954–7963 32931654 10.1002/cam4.3427 PMC7643676

[67] Wong D, Luo P, Oldfield LE et al (2024) Early cancer detection in Li–Fraumeni syndrome with cell-free DNA. Cancer Discov 14:104–119 37874259 10.1158/2159-8290.CD-23-0456 PMC10784744

[68] Llach J, Pellisé M, Monahan K (2022) Lynch syndrome; towards more personalized management? Best Pract Res Clin Gastroenterol 58:101790 35988964 10.1016/j.bpg.2022.101790

[69] Ligtenberg MJ, Kuiper RP, Geurts van Kessel A et al (2013) EPCAM deletion carriers constitute a unique subgroup of Lynch syndrome patients. Familial Cancer 12:169–174 23264089 10.1007/s10689-012-9591-x

[70] Dominguez-Valentin M, Sampson JR, Seppälä TT et al (2020) Cancer risks by gene, age, and gender in 6350 carriers of pathogenic mismatch repair variants: findings from the Prospective Lynch Syndrome Database. Genet Med 22:15–25 31337882 10.1038/s41436-019-0596-9 PMC7371626

[71] Ten Broeke SW, van der Klift HM, Tops CM et al (2018) Cancer risks for PMS2-associated Lynch syndrome. J Clin Oncol 36:2961–2968 30161022 10.1200/JCO.2018.78.4777 PMC6349460

[72] Goldberg M, Bell K, Aronson M et al (2017) Association between the Lynch syndrome gene *MSH2* and breast cancer susceptibility in a Canadian familial cancer registry. J Med Genet 54:742–746 28779004 10.1136/jmedgenet-2017-104542

[73] Espenschied CR, LaDuca H, Li S et al (2017) Multigene panel testing provides a new perspective on Lynch syndrome. J Clin Oncol 35:2568–2575. 10.1200/JCO.2016.71.9260 28514183 10.1200/JCO.2016.71.9260 PMC7186580

[74] Roberts ME, Jackson SA, Susswein LR et al (2018) MSH6 and PMS2 germ-line pathogenic variants implicated in Lynch syndrome are associated with breast cancer. Genet Med 20:1167–1174. 10.1038/gim.2017.254 29345684 10.1038/gim.2017.254 PMC6051923

[75] Leitlinienprogramm Onkologie (Deutsche Krebsgesellschaft DK, AWMF) (2024) S3-Leitlinie Endometriumkarzinom, Langversion 3.0

[76] Crosbie EJ, Ryan NA, Arends MJ et al (2019) The Manchester International Consensus Group recommendations for the management of gynecological cancers in Lynch syndrome. Genet Med 21:2390–2400 30918358 10.1038/s41436-019-0489-y PMC6774998

[77] Møller P, Seppälä TT, Ahadova A et al (2023) Dominantly inherited micro-satellite instable cancer–the four Lynch syndromes-an EHTG, PLSD position statement. Hered Cancer Clin Pract 21:19 37821984 10.1186/s13053-023-00263-3 PMC10568908

[78] Seppälä T, Latchford A, Negoi I et al (2021) European guidelines from the EHTG and ESCP for Lynch syndrome: an updated third edition of the Mallorca guidelines based on gene and gender. Br J Surg 108:484–498 34043773 10.1002/bjs.11902 PMC10364896

[79] Hüneburg R, Aretz S, Büttner R et al (2019) Empfehlungen zur Früherkennung, Risikoreduktion, Überwachung und Therapie bei Patienten mit Lynch-Syndrom. Z Gastroenterol 57:1309–1320 31739377 10.1055/a-1008-9827

[80] Burn J, Sheth H, Elliott F et al (2020) Cancer prevention with aspirin in hereditary colorectal cancer (Lynch syndrome), 10-year follow-up and registry-based 20-year data in the CAPP2 study: a double-blind, randomised, placebo-controlled trial. Lancet 395:1855–1863 32534647 10.1016/S0140-6736(20)30366-4 PMC7294238

[81] Robertson JC, Jorcyk CL, Oxford JT (2018) DICER1 syndrome: DICER1 mutations in rare cancers. Cancers (Basel) 10:20180515. 10.3390/cancers10050143 10.3390/cancers10050143 PMC5977116 29762508

[82] Schultze-Florey RE, Graf N, Vorwerk P et al (2013) DICER1 syndrome: a new cancer syndrome. Klin Padiatr 225:177–178. 10.1055/s-0033-1337976 23625684 10.1055/s-0033-1337976

[83] Schultz KAP, Stewart DR, Kamihara J, et al. (1993) DICER1 tumor predisposition. In: Adam MP, Feldman J, Mirzaa GM, et al. (eds) GeneReviews((R)), Seattle (WA) 24761742

[84] Blair VR, McLeod M, Carneiro F et al (2020) Hereditary diffuse gastric cancer: updated clinical practice guidelines. Lancet Oncol 21:e386–e397 32758476 10.1016/S1470-2045(20)30219-9 PMC7116190

[85] Roberts ME, Ranola JMO, Marshall ML et al (2019) Comparison of *CDH1* penetrance estimates in clinically ascertained families vs families ascertained for multiple gastric cancers. JAMA Oncol 5:1325–1331 31246251 10.1001/jamaoncol.2019.1208 PMC6604087

[86] Hansford S, Kaurah P, Li-Chang H et al (2015) Hereditary diffuse gastric cancer syndrome: *CDH1* mutations and beyond. JAMA Oncol 1:23–32 26182300 10.1001/jamaoncol.2014.168

[87] Leitlinienprogramm Onkologie (Deutsche Krebsgesellschaft DK, AWMF) (2025) Diagnostik und Therapie der Adenokarzinome des Magens und ösophagogastralen Übergangs, Langversion 3.0

[88] Varon RDI, Chrzanowska KH (1999) Nijmegen breakage syndrome. In: GeneReviews®[Internet] Seattle (WA): University of Washington, Seattle 1993–2025, [updated 2023 Nov 30] 20301355

[89] El Hasbaoui B, Elyajouri A, Abilkassem R, et al. (2020) Nijmegen breakage syndrome: case report and review of literature. Pan Afric Med J 35 10.11604/pamj.2020.35.85.14746 PMC7250236 32537088

[90] Bogdanova N, Feshchenko S, Schürmann P et al (2008) Nijmegen breakage syndrome mutations and risk of breast cancer. Int J Cancer 122:802–806 17957789 10.1002/ijc.23168

[91] Filipiuk A, Kozakiewicz A, Kośmider K et al (2022) Diagnostic and therapeutic approach to children with Nijmegen breakage syndrome in relation to development of lymphoid malignancies. Ann Agric Environ Med 29:207–214 35767752 10.26444/aaem/143541

[92] Hauke J, Horvath J, Groß E et al (2018) Gene panel testing of 5589 BRCA 1/2-negative index patients with breast cancer in a routine diagnostic setting: results of the German Consortium for Hereditary Breast and Ovarian Cancer. Cancer Med 7:1349–1358 29522266 10.1002/cam4.1376 PMC5911592

[93] Rusak B, Kluźniak W, Wokołorczyk D et al (2019) Allelic modification of breast cancer risk in women with an NBN mutation. Breast Cancer Res Treat 178:427–431 31410679 10.1007/s10549-019-05391-w

[94] Zhang G, Zeng Y, Liu Z et al (2013) Significant association between Nijmegen breakage syndrome 1 657del5 polymorphism and breast cancer risk. Tumor Biol 34:2753–2757 10.1007/s13277-013-0830-z 23765759

[95] Philpott C, Tovell H, Frayling IM et al (2017) The NF1 somatic mutational landscape in sporadic human cancers. Hum Genomics 11:13 28637487 10.1186/s40246-017-0109-3 PMC5480124

[96] Walker L, Thompson D, Easton D et al (2006) A prospective study of neurofibromatosis type 1 cancer incidence in the UK. Br J Cancer 95:233–238 16786042 10.1038/sj.bjc.6603227 PMC2360616

[97] Rasmussen SA, Yang Q, Friedman J (2001) Mortality in neurofibromatosis 1: an analysis using US death certificates. Am J Hum Genet 68:1110–1118 11283797 10.1086/320121 PMC1226092

[98] Howell SJ, Hockenhull K, Salih Z et al (2017) Increased risk of breast cancer in neurofibromatosis type 1: current insights. Breast Cancer Targets Ther. 10.2147/BCTT.S111397 10.2147/BCTT.S111397 PMC5573065 28860858

[99] Hu C, Hart SN, Gnanaolivu R et al (2021) A population-based study of genes previously implicated in breast cancer. N Engl J Med 384:440–451 33471974 10.1056/NEJMoa2005936 PMC8127622

[100] Evans DGR, Kallionpää RA, Clementi M et al (2020) Breast cancer in neurofibromatosis 1: survival and risk of contralateral breast cancer in a five country cohort study. Genet Med 22:398–406 31495828 10.1038/s41436-019-0651-6 PMC7000349

[101] Razavi P, Chang MT, Xu G et al (2018) The genomic landscape of endocrine-resistant advanced breast cancers. Cancer Cell 34(427–438):e426 10.1016/j.ccell.2018.08.008 PMC6327853 30205045

[102] Carton C, Evans DG, Blanco I et al (2023) ERN genturis tumour surveillance guidelines for individuals with neurofibromatosis type 1. EClinicalMedicine. 10.1016/j.eclinm.2022.101818 36684394 10.1016/j.eclinm.2022.101818 PMC9845795

[103] Hearle N, Schumacher V, Menko FH et al (2006) Frequency and spectrum of cancers in the Peutz-Jeghers syndrome. Clin Cancer Res 12:3209–3215 16707622 10.1158/1078-0432.CCR-06-0083

[104] Lim W, Olschwang S, Keller JJ et al (2004) Relative frequency and morphology of cancers in STK11 mutation carriers. Gastroenterology 126:1788–1794 15188174 10.1053/j.gastro.2004.03.014

[105] Van Lier M, Wagner A, Mathus-Vliegen E et al (2010) High cancer risk in Peutz-Jeghers syndrome: a systematic review and surveillance recommendations. Off J Am Coll Gastroenterol ACG 105:1258–1264 10.1038/ajg.2009.725 20051941

[106] Wagner A, Aretz S, Auranen A et al (2021) The management of Peutz-Jeghers syndrome: European hereditary tumour group (EHTG) guideline. J Clin Med 10:473 33513864 10.3390/jcm10030473 PMC7865862

[107] Pîrlog L-M, Pătrășcanu A-A, Militaru MS et al (2024) Insights into clinical disorders in Cowden syndrome: a comprehensive review. Medicina (Kaunas) 60:767 38792950 10.3390/medicina60050767 PMC11123368

[108] Pilarski R (2019) PTEN hamartoma tumor syndrome: a clinical overview. Cancers (Basel) 11:20190618. 10.3390/cancers11060844 10.3390/cancers11060844 PMC6627214 31216739

[109] Dragoo DD, Taher A, Wong VK et al (2021) PTEN hamartoma tumor syndrome/Cowden syndrome: genomics, oncogenesis, and imaging review for associated lesions and malignancy. Cancers (Basel) 13:3120 34206559 10.3390/cancers13133120 PMC8268822

[110] Gammon A, Jasperson K, Champine M (2016) Genetic basis of Cowden syndrome and its implications for clinical practice and risk management. Appl Clin Genet. 10.2147/TACG.S41947 27471403 10.2147/TACG.S41947 PMC4948690

[111] Pilarski R (2009) Cowden syndrome: a critical review of the clinical literature. J Genet Couns 18:13–27 18972196 10.1007/s10897-008-9187-7

[112] Pilarski R, Burt R, Kohlman W et al (2013) Cowden syndrome and the PTEN hamartoma tumor syndrome: systematic review and revised diagnostic criteria. J Natl Cancer Inst 105:1607–1616 24136893 10.1093/jnci/djt277

[113] Tan M-H, Mester JL, Ngeow J et al (2012) Lifetime cancer risks in individuals with germline PTEN mutations. Clin Cancer Res 18:400–407 22252256 10.1158/1078-0432.CCR-11-2283 PMC3261579

[114] Bubien V, Bonnet F, Brouste V et al (2013) High cumulative risks of cancer in patients with PTEN hamartoma tumour syndrome. J Med Genet 50:255–263 23335809 10.1136/jmedgenet-2012-101339

[115] Tischkowitz M, Colas C, Pouwels S et al (2020) Cancer surveillance guideline for individuals with PTEN hamartoma tumour syndrome. Eur J Hum Genet 28:1387–1393. 10.1038/s41431-020-0651-7 32533092 10.1038/s41431-020-0651-7 PMC7608293

[116] Magaña M, Landeta-Sa AP, López-Flores Y (2022) Cowden disease: a review. Am J Dermatopathol 44:705–717 36122333 10.1097/DAD.0000000000002234

[117] Takayama T, Muguruma N, Igarashi M et al (2023) Clinical guidelines for diagnosis and management of Cowden syndrome/*PTEN* hamartoma tumor syndrome in children and adults-secondary publication. J Anus Rectum Colon 7:284–300 37900693 10.23922/jarc.2023-028 PMC10600266

[118] Heaney RM, Farrell M, Stokes M et al (2017) Cowden syndrome: serendipitous diagnosis in patients with significant breast disease. Case series and literature review. Breast J 23:90–94 27886412 10.1111/tbj.12691

[119] Yehia L and Eng C (1993) PTEN hamartoma tumor syndrome. In: Adam MP, Feldman J, Mirzaa GM, et al. (eds) GeneReviews((R)). Seattle (WA) 20301661

[120] Kapačinskaitė M, Stratica N, Adomaitienė I et al (2024) A systematic review of Bannayan–Riley–Ruvalcaba syndrome. Sci Rep 14:21119 39256443 10.1038/s41598-024-71991-2 PMC11387762

[121] Witkowski L, Nichols KE, Jongmans M et al (2023) Germline pathogenic SMARCA4 variants in neuroblastoma. J Med Genet 60:987–992. 10.1136/jmg-2022-108854 36813544 10.1136/jmg-2022-108854 PMC10570933

[122] Connor YD, Miao D, Lin DI et al (2020) Germline mutations of SMARCA4 in small cell carcinoma of the ovary, hypercalcemic type and in SMARCA4-deficient undifferentiated uterine sarcoma: clinical features of a single family and comparison of large cohorts. Gynecol Oncol 157(1):106–114. 10.1016/j.ygyno.2019.10.031 31954538 10.1016/j.ygyno.2019.10.031 PMC7887697

[123] Ray-Coquard I, Morice P, Lorusso D et al (2018) Non-epithelial ovarian cancer: ESMO clinical practice guidelines for diagnosis, treatment and follow-up. Ann Oncol 29:iv1–iv18. 10.1093/annonc/mdy001 29697741 10.1093/annonc/mdy001

[124] Foulkes WD, Clarke BA, Hasselblatt M et al (2014) No small surprise - small cell carcinoma of the ovary, hypercalcaemic type, is a malignant rhabdoid tumour. J Pathol 233:209–214. 10.1002/path.4362 24752781 10.1002/path.4362

[125] Ramos P, Karnezis AN, Hendricks WP et al (2014) Loss of the tumor suppressor SMARCA4 in small cell carcinoma of the ovary, hypercalcemic type (SCCOHT). Rare Dis 2(1):e967148. 10.4161/2167549X.2014.967148 26942101 10.4161/2167549X.2014.967148 PMC4755243

[126] Herold N, Schmolling J, Ernst C et al (2023) Pathogenic germline variants in SMARCA4 and further cancer predisposition genes in early onset ovarian cancer. Cancer Med 12:15256–15260. 10.1002/cam4.6214 37345881 10.1002/cam4.6214 PMC10417158

[127] Nemes K BS, Bourdeaut F, et al. (2017) Rhabdoid tumor predisposition syndrome.In: GeneReviews® [Internet] Seattle (WA): University of Washington, Seattle 1993–2025. [Updated 2022 May 12] 29215836

[128] Curatolo P, Bombardieri R, Jozwiak S (2008) Tuberous sclerosis. Lancet 372:657–668. 10.1016/S0140-6736(08)61279-9 18722871 10.1016/S0140-6736(08)61279-9

[129] Northrup H, Aronow ME, Bebin EM et al (2021) Updated international tuberous sclerosis complex diagnostic criteria and surveillance and management recommendations. Pediatr Neurol 123:50–66. 10.1016/j.pediatrneurol.2021.07.011 34399110 10.1016/j.pediatrneurol.2021.07.011

[130] Celik H, Kefeli M, Cetinkaya M et al (2018) Perivascular epithelioid cell tumor (PEComa) of the uterine cervix in a patient with tuberous sclerosis complex: a literature review. Turk Patoloji Derg 34:82–86. 10.5146/tjpath.2014.01274 25110244 10.5146/tjpath.2014.01274

[131] Nishio N, Kido A, Minamiguchi S et al (2019) MR findings of uterine PEComa in patients with tuberous sclerosis: report of two cases. Abdom Radiol (NY) 44:1256–1260. 10.1007/s00261-019-01918-3 30778737 10.1007/s00261-019-01918-3

[132] Musella A, De Felice F, Kyriacou AK et al (2015) Perivascular epithelioid cell neoplasm (PEComa) of the uterus: a systematic review. Int J Surg 19(1–5):20150514. 10.1016/j.ijsu.2015.05.002 10.1016/j.ijsu.2015.05.002 25981307

[133] Izubuchi Y, Tanaka T (2025) PEComa-its clinical features, histopathology, and current therapy. Jpn J Clin Oncol 55:691–698. 10.1093/jjco/hyaf056 40336169 10.1093/jjco/hyaf056

